# Age‐related differences in functional network segregation in the context of sex and reproductive stage

**DOI:** 10.1002/hbm.26184

**Published:** 2022-12-21

**Authors:** Hannah K. Ballard, T. Bryan Jackson, Abigail C. Symm, Tracey H. Hicks, Jessica A. Bernard

**Affiliations:** ^1^ Texas A&M Institute for Neuroscience Texas A&M University College Station Texas USA; ^2^ Department of Psychological & Brain Sciences Texas A&M University College Station Texas USA

**Keywords:** aging, functional connectivity, menopause, network segregation, sex differences

## Abstract

Age is accompanied by differences in the organization of functional brain networks, which impact behavior in adulthood. Functional networks become less segregated and more integrated with age. However, sex differences in network segregation declines with age are not well‐understood. Further, network segregation in the context of female reproductive stage is relatively understudied, though unmasking such relationships would be informative for elucidating biological mechanisms that contribute to sex‐specific differences in aging. In the current work, we used data from the Cambridge Centre for Ageing and Neuroscience (Cam‐CAN) repository to evaluate differences in resting‐state network segregation as a product of sex and reproductive stage. Reproductive stage was categorized using the Stages of Reproductive Aging Workshop (STRAW+10) criteria. Replicating prior work, we investigated the following functional networks: auditory, cerebellar‐basal ganglia, cingulo‐opercular task control, default mode, dorsal attention, fronto‐parietal task control, salience, sensory somatomotor mouth, sensory somatomotor hand, ventral attention, and visual. First, our results mirror findings from previous work indicating that network segregation is lower with increasing age. Second, when analyzing associations between network segregation and age within each sex separately, we find qualitative differences between females and males. Finally, we report significant effects of reproductive stage on network segregation, though these findings are likely driven by age. Broadly, our results suggest that impacts of sex may be important to evaluate when investigating network segregation differences across adulthood, though further work is needed to determine the unique role of menopause and sex hormones on the organization of functional brain networks within aging females.

## Graphical abstract

Our results mirror findings from previous work indicating that functional network segregation is lower with increasing age. When analyzing associations between network segregation and age with respect to biological sex, we find qualitative differences between females and males. Finally, we report significant effects of reproductive stage on functional network segregation, though these findings are likely driven by age.

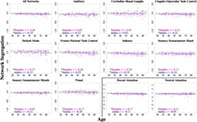

## 1 INTRODUCTION

With older age comes normative functional differences in both cognitive and motor domains (Harada et al., 2013; Leal & Yassa, 2014; Stöckel et al., 2017). These age‐related behavioral differences are linked to structural differences in brain volume (Bernard & Seidler, 2013; Raz & Rodrigue, 2006), as well as differences in the large‐scale organization of brain networks (Chan et al., 2014; Damoiseaux, 2017; King et al., 2018). Thus, understanding the factors that contribute to these brain‐behavior relationships is important for advancing care and improving quality of life for the aging population.

Many aging investigations focus on task‐based functional activation (Mirelman et al., 2017; Qin & Basak, 2020), though connectivity in the absence of a task is also informative for assessing differences in brain organization over the course of the adult lifespan (Ferreira & Busatto, 2013). The organization of functional brain networks is partially defined by network segregation, which represents greater within‐network connectivity strength relative to between‐network strength. Network segregation is thought to benefit specialized information processing and efficiency (Bullmore & Sporns, 2012; Wig, 2017) and is often evaluated in comparison to network integration, or dedifferentiation, which corresponds to greater connectivity between networks.

These measures of brain network organization are impacted by age. Young adults demonstrate multiple segregated functional networks with unique behavioral contributions (Power et al., 2011). However, network segregation is typically reduced in advanced age, resulting in increased integration/dedifferentiation of functional brain networks (Chan et al., 2014; Geerligs et al., 2015; Goh, 2011; Setton et al., 2022). Importantly, reduced network segregation, decreased modularity, and dedifferentiation are associated with worsened cognitive and motor performance (King et al., 2018; Kong et al., 2020). In fact, some work shows that network segregation mediates relationships between neurotransmitter systems and behavior in later life (Cassady et al., 2019). As such, the literature suggests that differences in the organization of functional brain networks across adulthood may be a key contributor to age‐related behavioral declines.

Notably, aging females are more affected by behavioral and brain differences, compared to males. For example, females demonstrate faster declines in global cognition and greater deficits in balance than males (Levine et al., 2021; Wolfson et al., 1994). Relatedly, females incur higher risk for age‐related diseases, such as Alzheimer's disease (Alzheimer's Association, 2021; Gao et al., 1998). These sex‐specific impacts of age may be related to biological characteristics, such as sex hormone changes with menopause. Menopause is characterized by the cessation of ovarian function, which initiates a decrease in estrogen and progesterone levels. Estrogen and progesterone have been shown to benefit cognition and brain health (Duka et al., 2000; Hara et al., 2015; Jacobs & D'Esposito, 2011; Singh & Su, 2013), and brain circuitry has been associated with hormonal fluctuations across the menstrual cycle (Jacobs et al., 2017; Pritschet et al., 2020). Thus, the loss of neuroprotective hormones with menopause may contribute to disproportionate aging impacts on older females.

However, research on network segregation with respect to sex differences and reproductive aging is lacking. The influence of menopause on the severity of functional declines in aging females is important to factor in when interrogating the origins of sex‐specific differences in aging. Such insight would offer important new avenues through which age‐related declines may be more effectively addressed. Given the increased incidence and severity of age‐related diseases (e.g., Alzheimer's disease) in females, this knowledge may also promote efforts in the early detection and treatment of disease progression. To address these gaps, we investigated differences in resting‐state network segregation between females and males, as well as between reproductive and postmenopausal females, across several functional brain networks. We also looked at associations between network segregation and age across sexes, replicating past research (Cassady et al., 2019; Chan et al., 2014), and within females and males separately.

In the interest of evaluating both cortical and subcortical network segregation, we included 10 cortical networks, as defined by Power et al. (2011), and one subcortical network, following Hausman et al. (2020). For the subcortical network, we included striatal seeds originally from Di Martino et al. (2008) and lobular cerebellar seeds that were created using the SUIT atlas (Diedrichsen, 2006; Diedrichsen et al., 2009), in lieu of using the undefined subcortical nodes/all‐encompassing subcortical network from Power et al. (2011). This approach allows us to investigate a more distinct and functionally‐specific subcortical network in the context of aging (i.e., a cerebellar‐basal ganglia network). In addition, we have previously shown differences in cerebellar‐basal ganglia connectivity between young and older adults (Hausman et al., 2020), further motivating the inclusion of this particular network in the current study. Striatal seeds were localized to the left hemisphere while cerebellar seeds were placed in the right hemisphere, given the known lateralization of cerebellar networks with cortical regions. These subcortical regions have reported age differences in connectivity, wherein older adults primarily show reduced resting‐state connectivity relative to young adults (Hausman et al., 2020), and are implicated in both motor and cognitive function (Bernard et al., 2017; Helie et al., 2013; King et al., 2019; Stoodley et al., 2012; Stoodley & Schmahmann, 2009). As such, subcortical networks may show differences in brain organization with advanced age, potentially contributing to age‐related functional declines. To follow‐up on prior work and further explore subcortical structures in the context of aging, we included this cerebellar‐basal ganglia network as an additional point of comparison in our analyses.

The current investigation was designed to answer several overarching questions. *Question 1*: Can we replicate prior findings showing reduced segregation in cortical networks with increasing age? Given that several studies have shown lower functional network segregation in advanced age (Chan et al., 2014; Geerligs et al., 2015; Goh, 2011; Setton et al., 2022), we predicted that similar age‐segregation associations would be present in the current sample. *Question 2*: Do we see the same age‐segregation relationships with subcortical structures? Considering the role of the cerebellum and basal ganglia in behaviors associated with age‐related declines (Bernard et al., 2017; Helie et al., 2013; King et al., 2019; Stoodley et al., 2012; Stoodley & Schmahmann, 2009), we anticipated that reduced network segregation with increased age would also emerge within this subcortical network. *Question 3*: Do patterns of network segregation declines with age differ between females and males? As females generally experience heavier burdens with older age (Alzheimer's Association, 2021; Gao et al., 1998; Levine et al., 2021; Wolfson et al., 1994), we predicted that reduced network segregation would be more pronounced in females, relative to males. *Question 4*: Does reproductive stage play a role in potential sex differences with age‐segregation relationships? With the benefits of sex hormones in mind (Duka et al., 2000; Hara et al., 2015; Jacobs & D'Esposito, 2011; Singh & Su, 2013), we expected to see greater differences in network segregation between female reproductive stages, as related to hormone loss with menopause, compared to age‐matched male controls. These questions are revisited when discussing the present findings.

## 2 MATERIALS AND METHODS

### 2.1 Study sample

Data was accessed through the Cambridge Centre for Ageing and Neuroscience (Cam‐CAN) repository (Shafto et al., 2014; Taylor et al., 2017). Data for this repository was gathered from a large sample of healthy adults, ranging from 18 to 88 years of age. We used raw structural and resting‐state magnetic resonance imaging (MRI) data, along with demographic variables including sex, age, and menstrual cycle characteristics. We initially acquired data for 652 participants; however, 54 of those participants were excluded for being left‐handed or for lacking handedness data. Handedness was assessed using the Edinburgh Handedness Inventory (Oldfield, 1971). This exclusionary criterion was applied to avoid the potential influence of brain organization differences between left‐handed and right‐handed individuals (Levy & Reid, 1978; Li et al., 2014). Additional individuals were excluded due to MRI data discrepancies, such as missing resting‐state scans (*n* = 4), significant motion artifacts that could not be corrected during image preprocessing (*n* = 1), and lack of full resting‐state volumes (*n* = 3). As a result, our initial sample consisted of 590 right‐handed participants (297 females).

### 2.2 Reproductive stage groupings

Our approach for categorizing females into reproductive, late perimenopausal, early postmenopausal, and late postmenopausal groups was replicated from previous work (Ballard et al., 2022). Here, a brief overview is provided. We used the Stages of Reproductive Aging Workshop Criteria (STRAW+10) to assign females to each reproductive stage group (Harlow et al., 2012). To distinguish between reproductive and late perimenopausal females, we used the reported length of menstrual cycles in days and number of days since last menstrual period. Females with 0–59 days for both variables were classified as reproductive, and females with 60–365 days were put in the late perimenopause group. Further, those within 1 year of their final menstrual period were also included in the late perimenopause group. To separate postmenopausal females into early and late groups, we used the number of years since final menstrual period. Females with 2–8 years since their final menstrual period were categorized as early postmenopausal, while those with 9+ years since their final menstrual period were assigned to the late postmenopause group.

Females lacking data for menstrual cycle characteristics were categorized by age cut‐offs (*n* = 24): 18–39 for reproductive, 40–49 for late perimenopausal, 55–70 for early postmenopausal, and 71 or older for late postmenopausal. Females ages 50–54, lacking menstrual cycle data, were excluded from final analyses (*n* = 5) due to variability in reproductive stage for females in this age range (Kato et al., 1998; Morabia & Costanza, 1998; Palmer et al., 2003). To minimize external influences on hormone levels and examine impacts of natural menopause, we excluded females with an intrauterine device (IUD) (*n* = 12), possible use of continuous birth control (*n* = 2), and history of hysterectomy (*n* = 1). Notably, we only excluded females who indicated a hysterectomy that were less than 71 years of age, given that those over the age of 71 with a hysterectomy (*n* = 7) are likely in a comparable hormonal state to naturally menopausal females of a similar age. The resulting groups from this staging approach were corroborated with subjective responses from females regarding the occurrence of menopause. For further details on our grouping approach, please refer to Ballard et al. (2022).

### 2.3 Age‐matching

To help account for the intrinsic impact of age on reproductive stage, we formed age‐matched male control groups to be used as an indirect reference for female groups. Each male was matched to a female using age, resulting in 1:1 age‐matching, along with two variables of quality assurance where necessary: number of outlier scans and maximum motion. Females and males did not significantly differ in either quality assurance variable (*ps* ≥ 0.09). When presented with multiple males of the same age, we chose the male with the number of outlier scans most similar to that of the female in question. If males of the same age also contained identical counts for outlier scans, we chose the male whose maximum motion value was closest to that of the female. In cases where there were more females than males for a particular age, the same approach using number of outlier scans and maximum motion was used to choose female matches. Un‐matched males and females were excluded from analyses (*n* = 156, 70 females); thus, our final sample consisted of 414 participants (207 females, ages 18–87, mean age 56.39 ± 18.80).

Our age‐matching method helps account for the natural linkage between age and menopause by facilitating sex comparisons between groups of equal age makeups and sample sizes. In fact, there is notable age overlap between the resulting female groups, even slightly between reproductive (ages 18–55) and late postmenopausal (ages 54–87) females. Characteristics of age‐matched groups are reported in Table 1. A graphical representation of this data is also available in Ballard et al. (2022), as groups were identical to those used in this prior work.

**TABLE 1:** Sample characteristics

Stage	Sample size	Mean age	Age range
Reproductive	71	35.04 ± 8.80	18–55
Late perimenopause	14	47.43 ± 4.70	40–58
Early postmenopause	24	56.50 ± 4.91	47–71
Late postmenopause	98	73.11 ± 7.74	54–87

*Note*: Sample size and age, in years, for each female reproductive group and relative male control group, after apply age‐matching exclusions. The numbers presented here correspond to both the female reproductive group and relative male control group, as 1:1 age‐matching resulted in equal age makeups and sample sizes between sexes.

### 2.4 Imaging analyses

A full overview of the study parameters and sample demographics for the Cam‐CAN repository can be found in Taylor et al. (2017) and Shafto et al. (2014). For our analyses, we used raw T1 MPRAGE structural scans and raw resting‐state EPI scans. The following parameters were used to collect resting‐state data: 8 min and 30 s of acquisition using a 3T Siemens TimTrio, 3 × 3 × 4.4 mm voxel size, and repetition time (TR) of 1.97 s.

Image preprocessing and analyses were performed using the CONN toolbox, version 19b (Whitfield‐Gabrieli & Nieto‐Castanon, 2012). We used the default preprocessing pipeline, which consists of realignment and unwarping with motion correction, centering to (0, 0, and 0) coordinates, slice‐timing correction, outlier detection using a 95th percentile threshold and the Artifact Rejection Toolbox (ART), segmentation of grey matter, white matter, and cerebrospinal fluid, normalization to MNI space, and spatial smoothing with a 5 mm full width at half‐maximum (FWHM) Gaussian kernel. A band‐pass filter of 0.008–0.09 Hz was applied to denoise data. The threshold for global‐signal *z*‐values was set at 3, while the motion correction threshold was set at 0.5 mm. After being de‐spiked during denoising to adhere to the global mean, six‐axis motion data and frame‐wise outliers were included as first‐level covariates.

MNI coordinates for each cortical node were retrieved from Cassady et al. (2019) (originally derived from Power et al. (2011)). The subcortical network included 20 nodes extracted from Hausman et al. (2020); basal ganglia seeds were originally taken from Di Martino et al. (2008) and cerebellar seeds were determined via the SUIT atlas (Diedrichsen, 2006; Diedrichsen et al., 2009). Combining 214 nodes across 10 cortical networks (Cassady et al., 2019; Power et al., 2011) and 20 subcortical nodes for the cerebellar‐basal ganglia network (Di Martino et al., 2008; Diedrichsen, 2006; Diedrichsen et al., 2009; Hausman et al., 2020), our final set of ROIs contained 234 nodes across 11 networks (Supplementary Table 1). MNI coordinates for each node were translated to voxel coordinates, which were subsequently used to create spherical seeds with 3.5 mm diameters in FSL (Jenkinson et al., 2012). Seeds were then treated as ROIs, and first‐level ROI‐to‐ROI relationships, thresholded at *p* (FDR‐corrected) < 0.05, were evaluated with a bivariate correlation approach.

Next, we looked at group differences between female reproductive stages, as well as age‐matched male controls, to investigate relationships with age and effects of sex and reproductive stage on network segregation. Replicating the approach of Chan et al. (2014) and Cassady et al. (2019), network segregation values were determined using Equation (1) below.

(1) $$\text{Network segregation}=\frac{{\bar{z}}_{w}-{\bar{z}}_{b}}{{\bar{z}}_{w}}.$$

Pearson's correlation coefficients were first transformed via Fisher's *r*‐to‐*z* conversion (Zar, 1996). In Equation (1) (Cassady et al., 2019; Chan et al., 2014), ${\bar{z}}_{w}$ corresponds to the mean correlation between all individual ROIs within a specific network, and ${\bar{z}}_{b}$ represents the mean correlation between the ROIs of a specific network and all remaining ROIs of other networks. Thus, all 234 nodes across the 11 networks included here were first considered at the ROI‐level before calculating correlation averages for each pre‐determined set of ROIs that indexed a particular functional network. The sign of these correlations was preserved during the transform process; thus, participants with more negative correlations of a higher value may have received an overall negative score for network segregation, depending on the signs of their within‐network and between‐network values as well as the relative weights of these values. Imaging analyses for the current work were carried out using the resources provided by the Texas A&M High Performance Research Computing organization.

### 2.5 Experimental design and statistical analyses

Following Chan et al. (2014) and Cassady et al. (2019), we first investigated relationships between mean network segregation (computed across all networks) and age, as well as age and segregation of each individual network, using Pearson's correlations. This was carried out with the whole sample. We then completed these analyses within females and males, separately, to elucidate potential sex‐specific differences in age‐segregation relationships. Finally, to reduce multiple comparisons, we used 2 × 2 between‐subjects ANOVAs to evaluate potential effects of sex (females vs. males) and reproductive stage (reproductive vs. late postmenopausal) on mean network segregation and segregation of individual networks.

Consistent with prior work (Cassady et al., 2019; Chan et al., 2014), segregation values above or below three standard deviations from the mean were excluded from final analyses. For analyses considering mean network segregation, exclusions were based on the overall segregation average across all networks, whereas exclusions relative to individual network means were applied to analyses that evaluated each network separately. For the cerebellar‐basal ganglia network, one extreme outlier with a segregation value of 697.86, corresponding to 20 standard deviations above the original network mean, was removed. To fairly screen for true outliers in the cerebellar‐basal ganglia network, this extreme outlier was removed before performing subsequent exclusions. Values above or below three standard deviations from the adjusted network mean, after removing the extreme outlier, were also excluded as outliers for the cerebellar‐basal ganglia network. All statistical analyses were conducted in R programming software.

## 3 RESULTS

### 3.1 Age correlations

**FIGURE 1:**
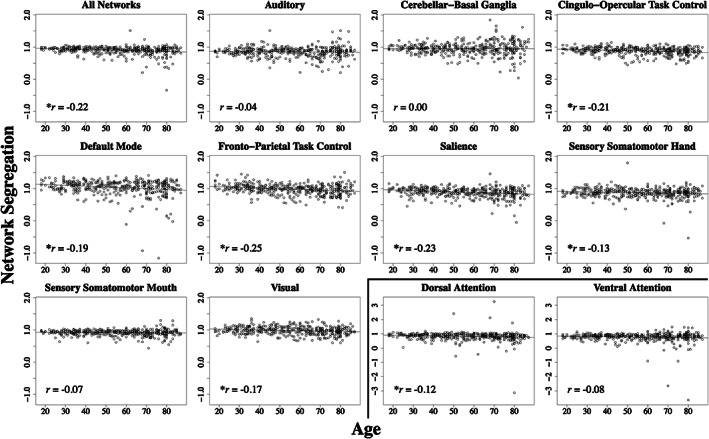
Relationships between age and network segregation across all participants. Distributions for each individual network and the mean across all networks. Each plot includes a linear regression line and the associated correlation value. *Significant *P*_fdr_ value after correcting for multiple comparisons (at least <.05). Two networks (dorsal attention and ventral attention) were plotted on a separate scale from all remaining networks for interpretability

Detailed results for network segregation and age correlations are reported in Table 2. When considering age and mean network segregation (computed across all networks), there is a significant correlation (*p* < .001) such that increased age is associated with lower overall network segregation across participants (Figure 1; Table 2). Considering each network individually, a significant correlation between age and network segregation emerges for 7 out of the 11 networks investigated after applying multiple comparisons corrections with a False Discovery Rate (FDR) approach: cingulo‐opercular task control, default mode, dorsal attention, fronto‐parietal task control, salience, sensory somatomotor hand, and visual. Each of these individual network correlations indicate lower network segregation with increased age (Figure 1; Table 2).

**TABLE 2:** Pearson's correlations between age and network segregation across the whole sample

Network	*T*	*Df*	*P*_raw_	*P*_fdr_	*R*
Whole sample					
All networks	−4.60	408	<0.001***	<0.001***	−0.22
Auditory	−0.90	410	0.367	–	−0.04
Cerebellar‐basal ganglia	0.06	408	0.950	–	0.00
Cingulo‐opercular task control	−4.42	405	<0.001***	<0.001***	−0.21
Default mode	−3.90	410	<0.001***	<0.001***	−0.19
Dorsal attention	−2.53	411	0.012*	0.018*	−0.12
Fronto‐parietal task control	−5.29	405	<0.001***	<0.001***	−0.25
Salience	−4.87	408	<0.001***	<0.001***	−0.23
Sensory somatomotor hand	−2.58	411	0.010*	0.017*	−0.13
Sensory somatomotor mouth	−1.36	406	0.175	–	−0.07
Ventral attention	−1.71	410	0.089	–	−0.08
Visual	−3.55	407	<0.001***	<0.001***	−0.17

*Note*: *P*_raw_: Un‐corrected p values; *P*_fdr_: Adjusted *p*‐values after FDR multiple comparisons corrections.

* *p* < .05,

** *p*< .01,

*** *p* < .001.

**FIGURE 2:**
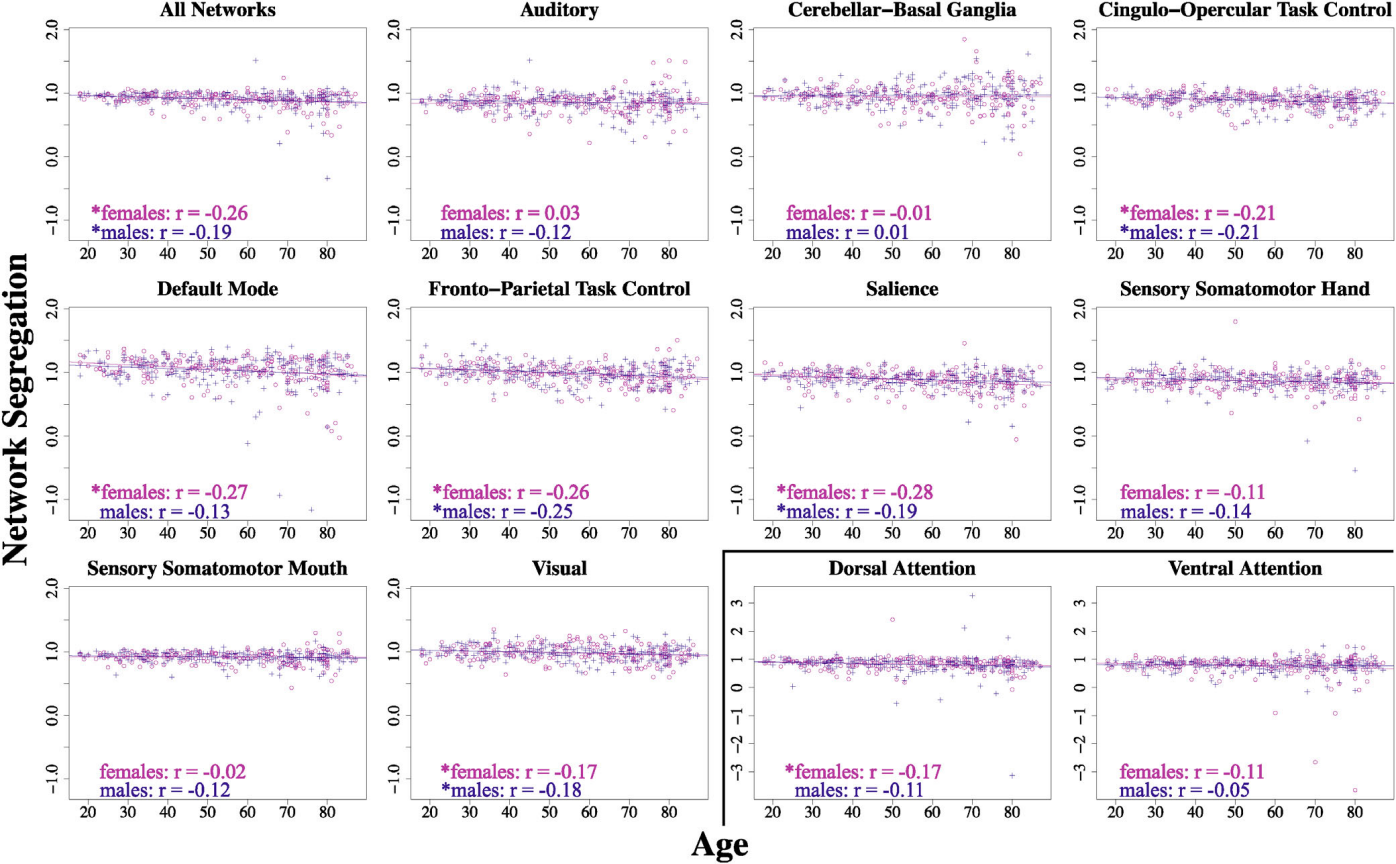
Relationships between age and network segregation, separated by sex. Distributions for each individual network and the mean across all networks. Females are plotted in purple and males in blue. Each plot includes linear regression lines and associated correlation values. *Significant *P*_fdr_ value after correcting for multiple comparisons (at least <.05). Two networks (dorsal attention and ventral attention) were plotted on a separate scale from other networks for interpretability

Correlations in females only reveal that older females exhibit lower segregation in almost all of the same networks as the whole sample: cingulo‐opercular task control, default mode, dorsal attention, fronto‐parietal task control, salience, and visual (Figure 2; Table 3). Further, females show a significant negative relationship between age and mean network segregation (*p* < .001). In contrast to the whole sample results, females do not demonstrate a significant correlation between age and segregation of the sensory somatomotor hand network (*p* = .124).

**TABLE 3:** Pearson's correlations between age and network segregation within each sex separately

Network	*T*	*Df*	*P*_raw_	*P*_fdr_	*R*
Females only					
All networks	−3.84	202	<0.001***	<0.001***	−0.26
Auditory	0.39	203	0.694	–	0.03
Cerebellar‐basal ganglia	−0.09	204	0.925	–	−0.01
Cingulo‐opercular task control	−3.13	202	0.002**	0.005**	−0.21
Default mode	−4.03	203	<0.001***	<0.001***	−0.27
Dorsal attention	−2.44	204	0.016*	0.027*	−0.17
Fronto‐parietal task control	−3.88	202	<0.001***	<0.001***	−0.26
Salience	−4.12	203	<0.001***	<0.001***	−0.28
Sensory somatomotor hand	−1.54	205	0.124	–	−0.11
Sensory somatomotor mouth	−0.28	202	0.779	–	−0.02
Ventral attention	−1.58	204	0.116	–	−0.11
Visual	−2.50	202	0.013*	0.027*	−0.17
Males only					
All networks	−2.78	204	0.006**	0.022*	−0.19
Auditory	−1.78	205	0.077	–	−0.12
Cerebellar‐basal ganglia	0.19	202	0.853	–	0.01
Cingulo‐opercular task control	−3.11	201	0.002**	0.013*	−0.21
Default mode	−1.91	205	0.058	–	−0.13
Dorsal attention	−1.58	205	0.116	–	−0.11
Fronto‐parietal task control	−3.59	201	<0.001***	0.005**	−0.25
Salience	−2.70	203	0.007**	0.022*	−0.19
Sensory somatomotor hand	−2.08	204	0.038*	0.077	−0.14
Sensory somatomotor mouth	−1.76	202	0.079	–	−0.12
Ventral attention	−0.66	204	0.510	–	−0.05
Visual	−2.54	203	0.012*	0.028*	−0.18

* *p* < .05,

** *p* < .01,

*** *p* < .001.

In age‐matched male controls, we see broadly similar associations between age and network segregation, though some differences emerge as well (Figure 2; Table 3). Mirroring females, male controls demonstrate lower segregation with increased age across all networks as well as within the cingulo‐opercular task control, fronto‐parietal task control, salience, and visual networks. However, unique to males, a negative association between age and network segregation is also present for the sensory somatomotor hand network, though this correlation does not survive multiple comparisons correction (*p* = .077). Interestingly, males do not demonstrate significant correlations between age and network segregation for the default mode and dorsal attention networks; thus, those relationships are unique to females. Overall, it seems that though some similarities exist between females and males, a few sex‐specific differences in functional network segregation declines with age are present in the current sample.

To follow‐up on these results and test potential interactions between sex and age, we then performed a series of ANCOVAs (Table 4). Each ANCOVA included sex and age, as well as the interaction between these variables, as factors with respect to network segregation. In brief, we found a main effect of age for the mean across all networks and for several individual networks after multiple comparisons corrections: cingulo‐opercular task control, default mode, fronto‐parietal task control, salience, and visual, which parallels the whole‐sample results. A significant effect of age was also observed for the ventral attention network, though this effect did not survive multiple comparisons correction (*p* = .072). Importantly, main effects of sex and interactions between sex and age were not revealed in any case with this particular series of analyses, standing in contrast to our prior within‐sex correlation results.

**TABLE 4:** ANCOVA results for sex‐age interactions

Effect	Dfn	Dfd	*F*	*P*_raw_	*P*_fdr_	Effect size
Sex (female vs. male) × age ANCOVAs						
All networks						
Age	1	406	12.16	<0.001***	0.002**	0.03
Sex	1	406	0.02	0.885	–	0.00
Age:sex	1	406	0.12	0.726	–	0.00
Auditory						
Age	1	408	0.17	0.680	–	0.00
Sex	1	408	2.71	0.101	–	0.01
Age:sex	1	408	2.21	0.138	–	0.01
Cerebellar‐basal ganglia						
Age	1	406	0.01	0.925	–	0.00
Sex	1	406	0.01	0.926	–	0.00
Age:sex	1	406	0.04	0.842	–	0.00
Cingulo‐opercular task control						
Age	1	403	9.81	0.002**	0.004**	0.02
Sex	1	403	0.00	0.944	–	0.00
Age:sex	1	403	0.00	0.969	–	0.00
Default mode						
Age	1	408	10.92	0.001**	0.003**	0.03
Sex	1	408	0.77	0.382	–	0.00
Age:sex	1	408	0.62	0.431	–	0.00
Dorsal attention						
Age	1	409	2.52	0.114	–	0.01
Sex	1	409	0.00	0.997	–	0.00
Age:sex	1	409	0.08	0.781	–	0.00
Fronto‐parietal task control						
Age	1	403	15.65	<0.001***	<0.001***	0.04
Sex	1	403	0.00	0.948	–	0.00
Age:sex	1	403	0.10	0.750	–	0.00
Salience						
Age	1	406	19.16	<0.001***	<0.001***	0.05
Sex	1	406	0.77	0.380	–	0.00
Age:sex	1	406	1.74	0.188	–	0.00
Sensory somatomotor hand						
Age	1	409	2.18	0.141	–	0.01
Sex	1	409	0.30	0.581	–	0.00
Age:sex	1	409	0.24	0.624	–	0.00
Sensory somatomotor mouth						
Age	1	404	0.09	0.762	–	0.00
Sex	1	404	0.89	0.347	–	0.00
Age:sex	1	404	0.85	0.357	–	0.00
Ventral attention						
Age	1	408	4.15	0.042*	0.072	0.01
Sex	1	408	0.73	0.393	–	0.00
Age:sex	1	408	1.38	0.241	–	0.00
Visual						
Age	1	405	7.22	0.008**	0.015*	0.02
Sex	1	405	0.00	0.963	–	0.00
Age:sex	1	405	0.07	0.797	–	0.00

* *p* < .05,

** *p* < .01,

*** *p* < .001.

### 3.2 Reproductive stage comparisons

**FIGURE 3:**
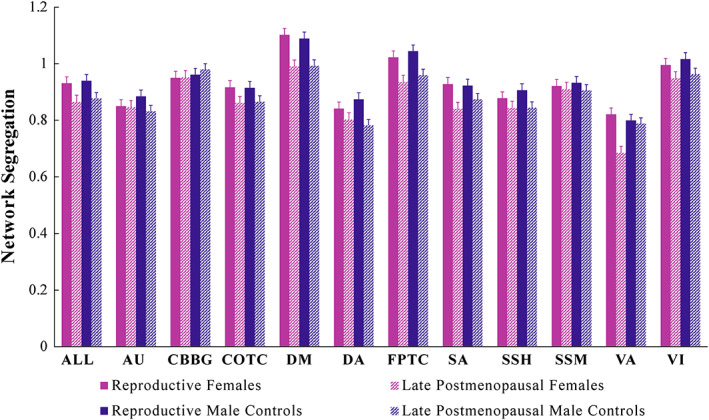
Network segregation by stage (reproductive vs. late postmenopausal/relative controls) and sex (female vs. male). Average segregation value per group for individual networks and the mean across all networks. Error bars depict standard error. ALL, mean across all networks; AU, auditory; CBBG, cerebellar‐basal ganglia; COTC, cingulo‐opercular task control; DM, default mode; DA, dorsal attention; FPTC, fronto‐parietal task control; SA, salience; SSH, sensory somatomotor hand; SSM, sensory somatomotor mouth; VA, ventral attention; VI, visual

An overview of reproductive stage and sex effects on network segregation is provided in Table 5. An effect of stage (reproductive vs. late postmenopausal/relative controls) was significant across networks when considered together (*p* < .001) as well as within six individual networks after correcting for multiple comparisons: cingulo‐opercular task control, default mode, fronto‐parietal task control, salience, sensory somatomotor hand, and visual. Notably, however, the effect of sex (females vs. males) was not statistically significant across networks, both on average and for the individual networks considered here. In addition, interactions between reproductive stage and sex were not significant for any networks. As such, given the lack of sex effects or interactions between stage and sex, this series of analyses suggests that reproductive stage within females does not differentially impact functional network segregation beyond the impacts of age more generally. The consistent significance of main effects for reproductive stage across both sexes may be more directly attributed to age alone (Figure 3). However, exploratory analyses with the reproductive and early postmenopause groups indicate that sex‐specific differences in network segregation, with respect to reproductive stage, may be present during the mid‐life transition to menopause (see Supplementary Table 2). This may suggest that the menopausal transition is particularly important for network dynamics, but in later life once hormones reach a more stable low state, sex differences are no longer present. However, we would note that this is exploratory and speculative.

**TABLE 5:** Between‐subjects ANOVA results

Effect	Dfn	Dfd	*F*	*P*_raw_	*P*_fdr_	Effect size
2 (reproductive vs. late postmenopausal) × 2 (female vs. male) ANOVAs						
All networks						
Stage	1	330	18.29	<0.001***	<0.001***	0.05
Sex	1	330	0.53	0.466	–	0.00
Stage:sex	1	330	0.01	0.919	–	0.00
Auditory						
Stage	1	332	2.33	0.128	–	0.01
Sex	1	332	0.11	0.736	–	0.00
Stage:sex	1	332	1.74	0.188	–	0.01
Cerebellar‐basal ganglia						
Stage	1	330	0.22	0.643	–	0.00
Sex	1	330	0.85	0.359	–	0.00
Stage:sex	1	330	0.14	0.711	–	0.00
Cingulo‐opercular task control						
Stage	1	329	18.11	<0.001***	<0.001***	0.05
Sex	1	329	0.02	0.889	–	0.00
Stage:sex	1	329	0.08	0.773	–	0.00
Default mode						
Stage	1	332	13.27	<0.001***	<0.001***	0.04
Sex	1	332	0.02	0.896	–	0.00
Stage:sex	1	332	0.08	0.777	–	0.00
Dorsal attention						
Stage	1	333	3.27	0.071	–	0.01
Sex	1	333	0.00	0.951	–	0.00
Stage:sex	1	333	0.55	0.459	–	0.00
Fronto‐parietal task control						
Stage	1	328	25.15	<0.001***	<0.001***	0.07
Sex	1	328	1.87	0.173	–	0.01
Stage:sex	1	328	0.01	0.930	–	0.00
Salience						
Stage	1	331	15.96	<0.001***	<0.001***	0.05
Sex	1	331	1.02	0.313	–	0.00
Stage:sex	1	331	1.30	0.254	–	0.00
Sensory somatomotor hand						
Stage	1	334	7.93	0.005**	0.009**	0.02
Sex	1	334	0.57	0.450	–	0.00
Stage:sex	1	334	0.66	0.417	–	0.00
Sensory somatomotor mouth						
Stage	1	329	3.01	0.084	–	0.01
Sex	1	329	0.03	0.871	–	0.00
Stage:sex	1	329	0.56	0.456	–	0.00
Ventral attention						
Stage	1	332	2.78	0.096	–	0.01
Sex	1	332	1.38	0.241	–	0.00
Stage:sex	1	332	2.01	0.157	–	0.01
Visual						
Stage	1	329	14.27	<0.001***	<0.001***	0.04
Sex	1	329	1.91	0.168	–	0.01
Stage:sex	1	329	0.04	0.849	–	0.00

*p < .05,

** *p* < .01,

*** *p* < .001.

## 4 DISCUSSION

In the current study, we investigated age‐related differences in functional network segregation in the context of sex and female reproductive stage. Following previous work (Cassady et al., 2019; Chan et al., 2014), we examined 10 cortical networks (Power et al., 2011) and also included an additional subcortical network (Di Martino et al., 2008; Diedrichsen, 2006; Diedrichsen et al., 2009; Hausman et al., 2020). We first replicated past work showing lower network segregation with higher age across participants, as well as within females and males separately, answering *Question 1* and consistent with our predictions. Contrary to what we predicted for *Question 2* however, we did not find significant age‐segregation relationships with the subcortical network. Next, when evaluating relationships between age and network segregation within each sex separately, we found distinct patterns between females and males, along with some similarities, which partially supports our predictions related to *Question 3*. However, this conclusion was subsequently challenged by the lack of significant sex effects or sex‐age interactions with our ANCOVAs, leaving *Question 3* open for further investigation. Finally, we explored effects of sex and reproductive stage on network segregation; these tests revealed significant effects of reproductive stage on functional network segregation, while effects of sex and stage by sex interactions were null. These particular results leave *Question 4* unanswered, as more work is needed to define the role of menopause in sex differences with age‐related network segregation declines. Our results are further discussed in the context of the relevant literature below.

### 4.1 Resting‐state network segregation and aging

When looking at associations between mean network segregation and age across all participants, we find a significant correlation in the negative direction, indicating lower overall network segregation with increased age. This parallels the overarching theme in the literature, wherein older age is associated with lower network segregation and, in turn, greater integration between networks (Cassady et al., 2019; Chan et al., 2014; Geerligs et al., 2015; Grady et al., 2016; King et al., 2018; Spreng et al., 2016). As functional network segregation is associated with specialized information processing and efficiency (Bullmore & Sporns, 2012), as well as successful cognition and motor function (King et al., 2018; Kong et al., 2020), age‐related differences in network segregation, as observed here and in prior work, may contribute to functional declines in the aging population. This shift from network segregation to integration, or dedifferentiation, may represent a compensatory mechanism for the natural depreciation of brain function with age, in turn, impacting functional performance.

Relatedly, when investigating age‐segregation relationships within individual networks (using the whole sample), we found that segregation was lower with higher age in 7 out of 11 functional networks after correcting for multiple comparisons with an FDR approach: cingulo‐opercular task control, default mode, dorsal attention, fronto‐parietal task control, salience, sensory somatomotor hand, and visual. This is broadly consistent with findings from Chan et al. (2014) and Cassady et al. (2019), who reported similar relationships in 8/10 and 5/10 networks, respectively. We observed age‐related associations with network segregation in regions responsible for several behavioral domains, such as adaptive task control, spontaneous cognition, and working memory (Andrews‐Hanna et al., 2010; Marek & Dosenbach, 2018; Wallis et al., 2015).

We did not observe significant correlations with age in 4 of the 11 networks after multiple comparisons correction: auditory, cerebellar‐basal ganglia, sensory somatomotor mouth, and ventral attention. This indicates that, contrary to our expectations, subcortical network segregation, at least in the cerebellar‐basal ganglia network, is not correlated with age. Notably, we investigated this network as a whole for the current investigation, though in our prior work, we did show some degree of functional dedifferentiation *within* the cerebellar‐basal ganglia network in older adults. That is, motor nodes became less strongly associated with one another as did nodes associated with cognitive networks and structures (Hausman et al., 2020). Here, in relation to other cortical networks, we did not see any age‐segregation associations for this specific subcortical network. As such, we suggest that within network dynamics change and show evidence for dedifferentiation subcortically, but this is distinct from broader global dynamics with cortical networks.

In addition, this demonstrates that networks responsible for attentional filtering (ventral attention) (Vossel et al., 2014) and the interpretation of sensory information (auditory and sensory somatomotor mouth) (Kayser et al., 2005; Small & Green, 2012) do not show the same lessened segregation in older age as the other networks investigated. However, this is unlike Cassady et al. (2019) who found significant age differences in functional segregation with the auditory and sensory somatomotor mouth networks, whereas we did not. Moreover, Chan et al. (2014) did not find segregation‐age associations in the salience network, while such a relationship was in fact observed here. Importantly, our study included 414 participants, whereas previous works had substantially smaller samples, which may partially explain some of the observed differences between our results and findings from the larger body of work on network segregation in older adulthood. However, the mixed findings across these studies collectively suggest that the relationships between segregation of these networks and age are certainly less reliable and robust than other cortical networks.

### 4.2 Sex‐specific differences in network segregation

When breaking down age and network segregation associations by sex, we find both similarities and differences in females and males. Both sexes exhibit lower segregation with older age in cingulo‐opercular task control, fronto‐parietal task control, salience, and visual networks, though females also demonstrate negative correlations with age in the default mode and dorsal attention networks while these relationships were not seen in males. Thus, females may endure greater consequences with age in respect to the organization and efficiency of these particular functional networks, which may contribute to the disproportionate impact of normative behavioral declines and age‐related disease on aging females, compared to males. Interestingly, the default mode network is strongly implicated in Alzheimer's disease pathology, which is more prevalent and severe in females (Alzheimer's Association, 2021; Greicius et al., 2004; Jones et al., 2011). Suppression of default mode network activity is associated with better performance on cognitive tasks (Anticevic et al., 2012). Consequently, the lack of default mode network segregation with age, specifically in females, may also reflect an inability to successfully inhibit default mode function during task‐positive processing, in turn, contributing to age‐related behavioral deficits.

On the other hand, males demonstrate unique age‐related declines in segregation of the sensory somatomotor hand network, whereas females do not, though this correlation was only trending towards significance after correcting for multiple comparisons. Interestingly, in our prior work examining resting‐state connectivity differences in cerebellar‐whole brain networks between female reproductive stages and age‐matched males, we found that male control groups exhibited greater differences in cerebellar‐somatosensory connectivity compared to female reproductive groups (Ballard et al., 2022). Specifically, late postmenopausal male controls showed lower connectivity between cerebellar regions associated with cognition (Crus I/II) and regions of the somatosensory cortex, relative to both reproductive male controls and female counterparts. Though this prior work used the same sample from the current investigation, our results here align with these previous findings and illustrate that connectivity differences in somatosensory regions, specific to males, stand when using an alternative analysis approach. In sum, our findings highlight that sex‐specific differences are important to consider when exploring relationships between age and the organization of functional brain networks.

When testing the statistical effects of sex and interactions between age and sex on network segregation in our ANCOVA series, we did not observe results that were indicative of sex‐specific differences. Rather, we observed several instances of a main effect of age on network segregation, akin to our whole‐sample results, though this age‐segregation relationship was not significantly modified by sex for any network variable. Thus, these subsequent findings challenge the conclusions drawn from our qualitative comparisons of the correlations in males and females alone and suggest that variability in network segregation declines with age may not be statistically or directly associated with biological sex.

In our pre‐ versus post‐menopause ANOVAs, reproductive stage influenced segregation for several networks while no effects of sex were observed. These findings may point to age effects, though impacts of sex hormone changes with menopause may also be at play and would be useful for better categorizing reproductive stage. Notably, in an exploratory analysis evaluating network segregation differences between reproductive and early postmenopausal groups, effects of sex and interactions between stage and sex begin to emerge in a few networks, though these effects are no longer statistically significant after correcting for multiple comparisons (Supplementary Table 2). While these results are not conclusive, this indicates that the transition to menopause and initial declines in sex hormones may be important to evaluate in the context of sex differences in aging outcomes, however after the menopausal transition there are fewer sex differences. As such, it may be the case that the dynamics of hormonal change are important in midlife, though we would caution that this is highly speculative at this point. We would also note that, without access to direct hormone data we cannot accurately tease out these potential influences on the present results. Further work assessing direct effects of sex hormones is needed to fully understand the impact of menopause on the brain in aging females.

To this point, the current body of work on network segregation in older age does not include sex‐specific analyses or comparisons between female reproductive stages (Cassady et al., 2019; Chan et al., 2014; Geerligs et al., 2015; Grady et al., 2016; King et al., 2018; Spreng et al., 2016). In fact, none of this work reports any analyses on network segregation differences between females and males. However, females endure more severe functional declines with age (Levine et al., 2021; Wolfson et al., 1994); thus, functional network segregation differences may contribute, at least in part, to the imbalance in aging trajectories between females and males. Results from the present work suggest that the effects of sex may be important and should be further interrogated when examining the organization of resting‐state networks with respect to aging, and more work including the potential influence of sex steroid hormones is needed in the context of female reproductive aging. Future investigations should include sex‐specific analyses and evaluate the effects of hormone changes with menopause when probing brain differences in older adulthood.

### 4.3 Exploratory analyses

An ongoing topic of discussion in the neuroimaging literature is the use of strict thresholds for motion correction in older adults, who typically have greater motion in the scanner. Some work suggests that a conservative threshold of 0.5 mm is sufficient to control for motion artifacts in older populations (Adams et al., 2019; Cassady et al., 2019; Cassady et al., 2021; Reagh et al., 2018), while other work recommends a more stringent threshold in general (Ciric et al., 2018; Power et al., 2014). However, recent work has also adopted liberal thresholds for motion correction in older adults (e.g., 0.9 mm) (Adams et al., 2021; Riphagen et al., 2020), challenging these standards. Thus, the current stance of the literature on this particular parameter is unclear and more work is needed to fully understand the implications of different motion correction thresholds, with particular respect to motion in older populations.

In the current study, we adopted a conservative threshold of 0.5 mm for motion correction, given that our older subjects, perhaps surprisingly, moved less than the young adults (mean motion‐age correlation: *p* < .001, *r* = −.44; maximum motion‐age correlation: *p* < .001, *r* = −.25). Prior work (e.g., Power et al., 2012; Van Dijk et al., 2012) suggests that increased motion may result in lower measures of connectivity and influence group difference analyses. Here, motion was higher in the young participants, suggesting that the decreased segregation measures cannot be explained solely by higher possible motion in the older individuals in our sample. In addition, the averages for mean motion (0.095 mm) and maximum motion (0.489 mm) across our cohort of 414 subjects were relatively low. However, in the interest of thoroughly exploring the effects of motion correction thresholds on the outcomes tested here, we include a supplementary analysis that replicates the present analyses using a stringent threshold of 0.2 mm. These findings are presented within the Supplementary results. In brief, we find that many of the significant effects from the initial analysis with 0.5 mm were reduced or completely lost after applying the 0.2 mm threshold; thus, these modified results are largely different from what previous work reports with respect to age‐segregation relationships (Cassady et al., 2019; Chan et al., 2014).

While this may indicate that a more stringent threshold tapers the risk of observing false positives, it could also suggest that the quality of data may be sacrificed as the information that remains after filtering with a more stringent approach may not be entirely representative of the true variability across subjects. Rather than offering premature speculations on these contrasting results, we present the new analysis in a supplement in order to contribute to ongoing conversations on the issue of motion artifacts, particularly in older adults.

### 4.4 Limitations

Though this investigation contributes to current advances in aging research, there are limitations worth noting. First, we lacked access to direct hormone data or data regarding consecutive cycle lengths for our reproductive stage categorizations. As a result, reproductive stage was characterized using self‐report menstrual information, and females undergoing hormone therapy or taking hormonal contraceptives may have been included in our sample. As the effects of menopause on functional network segregation may be more explicitly linked to hormone fluctuations, as opposed to broad reproductive stage differences, the lack of hormone data has limited our investigation. Moreover, given the lack of consecutive cycle data, our reproductive group may inherently include females in early perimenopause. Second, we did not evaluate behavioral performance. Therefore, interpretation of functional relevance is purely theoretical. Third, though a network‐level approach is informative for assessing differences in large‐scale brain organization, our network segregation measure lacks the nuance of a purely ROI‐based analysis that may yield added insight on a finer scale. Relatedly, the size (i.e., number of ROIs) per network varies and is, thereby, unequal across the functional networks included here, which may have impacted our comparisons. Finally, given that menopause is a product of aging, we cannot discount impacts of age on the current findings. However, female reproductive groups overlap in age and age‐matched male controls help to limit age impacts.

## 5 CONCLUSION

The current study, using data from the CamCAN repository, offers new insight into sex‐specific differences in the aging brain. Here, we evaluated the influence of sex and female reproductive stage on age‐related associations with functional network segregation. We provide preliminary evidence for distinct patterns of functional network segregation between females and males, through a qualitative lens, along with potential effects of reproductive stage, indicating that these biological factors may contribute to some degree to the differing aging trajectories between sexes. However, subsequent work is needed to determine the particular role of sex hormone fluctuations with menopause on brain differences within aging females. Such work is necessary to support findings from the present investigation and provide potential avenues through which age‐related declines may be alleviated. Further, given sex differences in non‐normative aging, elucidating relationships between menopause and the aging brain may also offer treatment alternatives for age‐related diseases, such as Alzheimer's disease and other dementias.

## CONFLICT OF INTEREST

The authors declare no competing financial or alternative interests.

## Supporting information


**Appendix S1:** Supporting information.

**Table S1:** Supporting information.

## Data Availability

All data incorporated in the present study was accessed through the Cam‐CAN online repository and is available for shared use at https://camcan-archive.mrc-cbu.cam.ac.uk/dataaccess/.

## References

[hbm26184-bib-0001] Adams, J. N. , Maass, A. , Berron, D. , Harrison, T. M. , Baker, S. L. , Thomas, W. P. , Stanfill, M. , & Jagust, W. J. (2021). Reduced repetition suppression in aging is driven by tau‐related hyperactivity in medial temporal lobe. The Journal of Neuroscience, 41, 3917–3931. 33731446 10.1523/JNEUROSCI.2504-20.2021 PMC8084317

[hbm26184-bib-0002] Adams, J. N. , Maass, A. , Harrison, T. M. , Baker, S. L. , & Jagust, W. J. (2019). Cortical tau deposition follows patterns of entorhinal functional connectivity in aging. eLife, 8, 1–22. 10.7554/eLife.49132 PMC6764824 31475904

[hbm26184-bib-0003] Alzheimer's Association . (2021). 2021 Alzheimer's disease facts and figures. Alzheimers Dement, 17, 327–406. 33756057 10.1002/alz.12328

[hbm26184-bib-0004] Andrews‐Hanna, J. R. , Reidler, J. S. , Huang, C. , & Buckner, R. L. (2010). Evidence for the default network's role in spontaneous cognition. Journal of Neurophysiology, 104, 322–335. 20463201 10.1152/jn.00830.2009 PMC2904225

[hbm26184-bib-0005] Anticevic, A. , Cole, M. W. , Murray, J. D. , Corlett, P. R. , Wang, X.‐J. , & Krystal, J. H. (2012). The role of default network deactivation in cognition and disease. Trends in Cognitive Sciences (Regular Ed), 16, 584–592. 10.1016/j.tics.2012.10.008 PMC3501603 23142417

[hbm26184-bib-0006] Ballard, H. K. , Jackson, T. B. , Hicks, T. H. , & Bernard, J. A. (2022). The association of reproductive stage with lobular cerebellar network connectivity across female adulthood. Neurobiology of Aging, 117, 139–150. 35738086 10.1016/j.neurobiolaging.2022.05.014 PMC10149146

[hbm26184-bib-0007] Bernard, J. A. , Russell, C. E. , Newberry, R. E. , Goen, J. R. M. , & Mittal, V. A. (2017). Patients with schizophrenia show aberrant patterns of basal ganglia activation: Evidence from ALE meta‐analysis. NeuroImage: Clinical, 14, 450–463. 28275545 10.1016/j.nicl.2017.01.034 PMC5328905

[hbm26184-bib-0008] Bernard, J. A. , & Seidler, R. D. (2013). Relationships between regional cerebellar volume and sensorimotor and cognitive function in young and older adults. Cerebellum, 12, 721–737. 23625382 10.1007/s12311-013-0481-z PMC3820158

[hbm26184-bib-0009] Bullmore, E. , & Sporns, O. (2012). The economy of brain network organization. Nature Reviews. Neuroscience, 13, 336–349. 22498897 10.1038/nrn3214

[hbm26184-bib-0010] Cassady, K. , Gagnon, H. , Lalwani, P. , Simmonite, M. , Foerster, B. , Park, D. , Peltier, S. J. , Petrou, M. , Taylor, S. F. , Weissman, D. H. , Seidler, R. D. , & Polk, T. A. (2019). Sensorimotor network segregation declines with age and is linked to GABA and to sensorimotor performance. NeuroImage, 186, 234–244. 30414983 10.1016/j.neuroimage.2018.11.008 PMC6338503

[hbm26184-bib-0011] Cassady, K. E. , Adams, J. N. , Chen, X. , Maass, A. , Harrison, T. M. , Landau, S. , Baker, S. , & Jagust, W. (2021). Alzheimer's pathology is associated with dedifferentiation of intrinsic functional memory networks in aging. Cerebral Cortex, 31, 4781–4793. 34037210 10.1093/cercor/bhab122 PMC8408467

[hbm26184-bib-0012] Chan, M. Y. , Park, D. C. , Savalia, N. K. , Petersen, S. E. , & Wig, G. S. (2014). Decreased segregation of brain systems across the healthy adult lifespan. Proceedings of the National Academy of Sciences of the United States of America, 111, E4997–E5006. 25368199 10.1073/pnas.1415122111 PMC4246293

[hbm26184-bib-0013] Ciric, R. , Rosen, A. F. G. , Erus, G. , Cieslak, M. , Adebimpe, A. , Cook, P. A. , Bassett, D. S. , Davatzikos, C. , Wolf, D. H. , & Satterthwaite, T. D. (2018). Mitigating head motion artifact in functional connectivity MRI. Nature Protocols, 13, 2801–2826. 30446748 10.1038/s41596-018-0065-y PMC8161527

[hbm26184-bib-0014] Damoiseaux, J. S. (2017). Effects of aging on functional and structural brain connectivity. NeuroImage, 160, 32–40. 28159687 10.1016/j.neuroimage.2017.01.077

[hbm26184-bib-0015] Di Martino, A. , Scheres, A. , Margulies, D. S. , Kelly, A. M. C. , Uddin, L. Q. , Shehzad, Z. , Biswal, B. , Walters, J. R. , Castellanos, F. X. , & Milham, M. P. (2008). Functional connectivity of human striatum: A resting state FMRI study. Cerebral Cortex, 18, 2735–2747. 18400794 10.1093/cercor/bhn041

[hbm26184-bib-0016] Diedrichsen, J. (2006). A spatially unbiased atlas template of the human cerebellum. NeuroImage, 33, 127–138. 16904911 10.1016/j.neuroimage.2006.05.056

[hbm26184-bib-0017] Diedrichsen, J. , Balsters, J. H. , Flavell, J. , Cussans, E. , & Ramnani, N. (2009). A probabilistic MR atlas of the human cerebellum. NeuroImage, 46, 39–46. 19457380 10.1016/j.neuroimage.2009.01.045

[hbm26184-bib-0018] Duka, T. , Tasker, R. , & McGowan, J. F. (2000). The effects of 3‐week estrogen hormone replacement on cognition in elderly healthy females. Psychopharmacology, 149, 129–139. 10805607 10.1007/s002139900324

[hbm26184-bib-0019] Ferreira, L. K. , & Busatto, G. F. (2013). Resting‐state functional connectivity in normal brain aging. Neuroscience and Biobehavioral Reviews, 37, 384–400. 23333262 10.1016/j.neubiorev.2013.01.017

[hbm26184-bib-0020] Gao, S. , Hendrie, H. C. , Hall, K. S. , & Hui, S. (1998). The relationships between age, sex, and the incidence of dementia and Alzheimer disease: A meta‐analysis. Archives of General Psychiatry, 55, 809–815. 9736007 10.1001/archpsyc.55.9.809

[hbm26184-bib-0021] Geerligs, L. , Renken, R. J. , Saliasi, E. , Maurits, N. M. , & Lorist, M. M. (2015). A brain‐wide study of age‐related changes in functional connectivity. Cerebral Cortex, 25, 1987–1999. 24532319 10.1093/cercor/bhu012

[hbm26184-bib-0022] Goh, J. O. S. (2011). Functional dedifferentiation and altered connectivity in older adults: Neural accounts of cognitive aging. Aging and Disease, 2, 30–48. 21461180 PMC3066008

[hbm26184-bib-0023] Grady, C. , Sarraf, S. , Saverino, C. , & Campbell, K. (2016). Age differences in the functional interactions among the default, frontoparietal control, and dorsal attention networks. Neurobiology of Aging, 41, 159–172. 27103529 10.1016/j.neurobiolaging.2016.02.020

[hbm26184-bib-0024] Greicius, M. D. , Srivastava, G. , Reiss, A. L. , & Menon, V. (2004). Default‐mode network activity distinguishes Alzheimer's disease from healthy aging: Evidence from functional MRI. Proceedings of the National Academy of Sciences of the United States of America, 101, 4637–4642. 15070770 10.1073/pnas.0308627101 PMC384799

[hbm26184-bib-0025] Hara, Y. , Waters, E. M. , McEwen, B. S. , & Morrison, J. H. (2015). Estrogen effects on cognitive and synaptic health over the lifecourse. Physiological Reviews, 95, 785–807. 26109339 10.1152/physrev.00036.2014 PMC4491541

[hbm26184-bib-0026] Harada, C. N. , Natelson Love, M. C. , & Triebel, K. L. (2013). Normal cognitive aging. Clinics in Geriatric Medicine, 29, 737–752. 24094294 10.1016/j.cger.2013.07.002 PMC4015335

[hbm26184-bib-0027] Harlow, S. D. , Gass, M. , Hall, J. E. , Lobo, R. , Maki, P. , Rebar, R. W. , Sherman, S. , Sluss, P. M. , de Villiers, T. J. , & STRAW + 10 Collaborative Group . (2012). Executive summary of the stages of reproductive aging workshop + 10: Addressing the unfinished agenda of staging reproductive aging. The Journal of Clinical Endocrinology and Metabolism, 97, 1159–1168. 22344196 10.1210/jc.2011-3362 PMC3319184

[hbm26184-bib-0028] Hausman, H. K. , Jackson, T. B. , Goen, J. R. M. , & Bernard, J. A. (2020). From synchrony to asynchrony: Cerebellar‐basal ganglia functional circuits in young and older adults. Cerebral Cortex, 30, 718–729. 10.1093/cercor/bhz121 31219563

[hbm26184-bib-0029] Helie, S. , Chakravarthy, S. , & Moustafa, A. A. (2013). Exploring the cognitive and motor functions of the basal ganglia: An integrative review of computational cognitive neuroscience models. Frontiers in Computational Neuroscience, 7, 174. 24367325 10.3389/fncom.2013.00174 PMC3854553

[hbm26184-bib-0030] Jacobs, E. , & D'Esposito, M. (2011). Estrogen shapes dopamine‐dependent cognitive processes: Implications for women's health. The Journal of Neuroscience, 31, 5286–5293. 21471363 10.1523/JNEUROSCI.6394-10.2011 PMC3089976

[hbm26184-bib-0031] Jacobs, E. G. , Weiss, B. , Makris, N. , Whitfield‐Gabrieli, S. , Buka, S. L. , Klibanski, A. , & Goldstein, J. M. (2017). Reorganization of functional networks in verbal working memory circuitry in early midlife: The impact of sex and menopausal status. Cerebral Cortex, 27, 2857–2870. 27178194 10.1093/cercor/bhw127 PMC6059144

[hbm26184-bib-0032] Jenkinson, M. , Beckmann, C. F. , Behrens, T. E. , Woolrich, M. W. , & Smith, S. M. (2012). FSL. Neuroimage, 62, 782–790. 21979382 10.1016/j.neuroimage.2011.09.015

[hbm26184-bib-0033] Jones, D. T. , Machulda, M. M. , Vemuri, P. , McDade, E. M. , Zeng, G. , Senjem, M. L. , Gunter, J. L. , Przybelski, S. A. , Avula, R. T. , Knopman, D. S. , Boeve, B. F. , Petersen, R. C. , & Jack, C. R. (2011). Age‐related changes in the default mode network are more advanced in Alzheimer disease. Neurology, 77, 1524–1531. 21975202 10.1212/WNL.0b013e318233b33d PMC3198977

[hbm26184-bib-0034] Kato, I. , Toniolo, P. , Akhmedkhanov, A. , Koenig, K. L. , Shore, R. , & Zeleniuch‐Jacquotte, A. (1998). Prospective study of factors influencing the onset of natural menopause. Journal of Clinical Epidemiology, 51, 1271–1276. 10086819 10.1016/s0895-4356(98)00119-x

[hbm26184-bib-0035] Kayser, C. , Petkov, C. I. , Augath, M. , & Logothetis, N. K. (2005). Integration of touch and sound in auditory cortex. Neuron, 48, 373–384. 16242415 10.1016/j.neuron.2005.09.018

[hbm26184-bib-0036] King, B. R. , van Ruitenbeek, P. , Leunissen, I. , Cuypers, K. , Heise, K. F. , Santos Monteiro, T. , Hermans, L. , Levin, O. , Albouy, G. , Mantini, D. , & Swinnen, S. P. (2018). Age‐related declines in motor performance are associated with decreased segregation of large‐scale resting state brain networks. Cerebral Cortex, 28, 4390–4402. 29136114 10.1093/cercor/bhx297 PMC6215458

[hbm26184-bib-0037] King, M. , Hernandez‐Castillo, C. R. , Poldrack, R. A. , Ivry, R. B. , & Diedrichsen, J. (2019). Functional boundaries in the human cerebellum revealed by a multi‐domain task battery. Nature Neuroscience, 22, 1371–1378. 31285616 10.1038/s41593-019-0436-x PMC8312478

[hbm26184-bib-0038] Kong, T. S. , Gratton, C. , Low, K. A. , Tan, C. H. , Chiarelli, A. M. , Fletcher, M. A. , Zimmerman, B. , Maclin, E. L. , Sutton, B. P. , Gratton, G. , & Fabiani, M. (2020). Age‐related differences in functional brain network segregation are consistent with a cascade of cerebrovascular, structural, and cognitive effects. Network Neuroscience, 4, 89–114. 32043045 10.1162/netn_a_00110 PMC7006874

[hbm26184-bib-0039] Leal, S. L. , & Yassa, M. A. (2014). Effects of aging on mnemonic discrimination of emotional information. Behavioral Neuroscience, 128, 539–547. 25150544 10.1037/bne0000011 PMC4196942

[hbm26184-bib-0040] Levine, D. A. , Gross, A. L. , Briceño, E. M. , Tilton, N. , Giordani, B. J. , Sussman, J. B. , Hayward, R. A. , Burke, J. F. , Hingtgen, S. , Elkind, M. S. V. , Manly, J. J. , Gottesman, R. F. , Gaskin, D. J. , Sidney, S. , Sacco, R. L. , Tom, S. E. , Wright, C. B. , Yaffe, K. , & Galecki, A. T. (2021). Sex differences in cognitive decline among US adults. JAMA Network Open, 4, e210169. 33630089 10.1001/jamanetworkopen.2021.0169 PMC7907956

[hbm26184-bib-0041] Levy, J. , & Reid, M. (1978). Variations in cerebral organization as a function of handedness, hand posture in writing, and sex. Journal of Experimental Psychology. General, 107, 119–144. 670905 10.1037//0096-3445.107.2.119

[hbm26184-bib-0042] Li, M. , Chen, H. , Wang, J. , Liu, F. , Long, Z. , Wang, Y. , Iturria‐Medina, Y. , Zhang, J. , Yu, C. , & Chen, H. (2014). Handedness‐ and hemisphere‐related differences in small‐world brain networks: A diffusion tensor imaging tractography study. Brain Connectivity, 4, 145–156. 24564422 10.1089/brain.2013.0211 PMC3961786

[hbm26184-bib-0043] Marek, S. , & Dosenbach, N. U. F. (2018). The frontoparietal network: Function, electrophysiology, and importance of individual precision mapping. Dialogues in Clinical Neuroscience, 20, 133–140. 30250390 10.31887/DCNS.2018.20.2/smarek PMC6136121

[hbm26184-bib-0044] Mirelman, A. , Maidan, I. , Bernad‐Elazari, H. , Shustack, S. , Giladi, N. , & Hausdorff, J. M. (2017). Effects of aging on prefrontal brain activation during challenging walking conditions. Brain and Cognition, 115, 41–46. 28433922 10.1016/j.bandc.2017.04.002

[hbm26184-bib-0045] Morabia, A. , & Costanza, M. C. (1998). International variability in ages at menarche, first livebirth, and menopause. World Health Organization collaborative study of neoplasia and steroid contraceptives. American Journal of Epidemiology, 148, 1195–1205. 9867266 10.1093/oxfordjournals.aje.a009609

[hbm26184-bib-0046] Oldfield, R. C. (1971). The assessment and analysis of handedness: The Edinburgh inventory. Neuropsychologia, 9, 97–113. 5146491 10.1016/0028-3932(71)90067-4

[hbm26184-bib-0047] Palmer, J. R. , Rosenberg, L. , Wise, L. A. , Horton, N. J. , & Adams‐Campbell, L. L. (2003). Onset of natural menopause in African American women. American Journal of Public Health, 93, 299–306. 12554590 10.2105/ajph.93.2.299 PMC1447734

[hbm26184-bib-0048] Power, J. D. , Barnes, K. A. , Snyder, A. Z. , Schlaggar, B. L. , & Petersen, S. E. (2012). Spurious but systematic correlations in functional connectivity MRI networks arise from subject motion. NeuroImage, 59, 2142–2154. 22019881 10.1016/j.neuroimage.2011.10.018 PMC3254728

[hbm26184-bib-0049] Power, J. D. , Cohen, A. L. , Nelson, S. M. , Wig, G. S. , Barnes, K. A. , Church, J. A. , Vogel, A. C. , Laumann, T. O. , Miezin, F. M. , Schlaggar, B. L. , & Petersen, S. E. (2011). Functional network organization of the human brain. Neuron, 72, 665–678. 22099467 10.1016/j.neuron.2011.09.006 PMC3222858

[hbm26184-bib-0050] Power, J. D. , Mitra, A. , Laumann, T. O. , Snyder, A. Z. , Schlaggar, B. L. , & Petersen, S. E. (2014). Methods to detect, characterize, and remove motion artifact in resting state fMRI. NeuroImage, 84, 320–341. 23994314 10.1016/j.neuroimage.2013.08.048 PMC3849338

[hbm26184-bib-0051] Pritschet, L. , Santander, T. , Taylor, C. M. , Layher, E. , Yu, S. , Miller, M. B. , Grafton, S. T. , & Jacobs, E. G. (2020). Functional reorganization of brain networks across the human menstrual cycle. NeuroImage, 220, 117091. 32621974 10.1016/j.neuroimage.2020.117091

[hbm26184-bib-0052] Qin, S. , & Basak, C. (2020). Age‐related differences in brain activation during working memory updating: An fMRI study. Neuropsychologia, 138, 107335. 31923524 10.1016/j.neuropsychologia.2020.107335 PMC7069667

[hbm26184-bib-0053] Raz, N. , & Rodrigue, K. M. (2006). Differential aging of the brain: Patterns, cognitive correlates and modifiers. Neuroscience and Biobehavioral Reviews, 30, 730–748. 16919333 10.1016/j.neubiorev.2006.07.001 PMC6601348

[hbm26184-bib-0054] Reagh, Z. M. , Noche, J. A. , Tustison, N. J. , Delisle, D. , Murray, E. A. , & Yassa, M. A. (2018). Functional imbalance of anterolateral entorhinal cortex and hippocampal dentate/CA3 underlies age‐related object pattern separation deficits. Neuron, 97, 1187–1198.e4. 29518359 10.1016/j.neuron.2018.01.039 PMC5937538

[hbm26184-bib-0055] Riphagen, J. M. , Schmiedek, L. , Gronenschild, E. H. B. M. , Yassa, M. A. , Priovoulos, N. , Sack, A. T. , Verhey, F. R. J. , & Jacobs, H. I. L. (2020). Associations between pattern separation and hippocampal subfield structure and function vary along the lifespan: A 7 T imaging study. Scientific Reports, 10, 7572. 32371923 10.1038/s41598-020-64595-z PMC7200747

[hbm26184-bib-0056] Setton, R. , Mwilambwe‐Tshilobo, L. , Girn, M. , Lockrow, A. W. , Baracchini, G. , Hughes, C. , Lowe, A. J. , Cassidy, B. N. , Li, J. , Luh, W.‐M. , Bzdok, D. , Leahy, R. M. , Ge, T. , Margulies, D. S. , Misic, B. , Bernhardt, B. C. , Stevens, W. D. , De Brigard, F. , Kundu, P. , … Spreng, R. N. (2022). Age differences in the functional architecture of the human brain. Cerebral Cortex, 1–21. 10.1093/cercor/bhac056 PMC9758585 35231927

[hbm26184-bib-0057] Shafto, M. A. , Tyler, L. K. , Dixon, M. , Taylor, J. R. , Rowe, J. B. , Cusack, R. , Calder, A. J. , Marslen‐Wilson, W. D. , Duncan, J. , Dalgleish, T. , Henson, R. N. , Brayne, C. , Matthews, F. E. , & Cam‐CAN . (2014). The Cambridge Centre for Ageing and Neuroscience (Cam‐CAN) study protocol: A cross‐sectional, lifespan, multidisciplinary examination of healthy cognitive ageing. BMC Neurology, 14, 204. 25412575 10.1186/s12883-014-0204-1 PMC4219118

[hbm26184-bib-0058] Singh, M. , & Su, C. (2013). Progesterone and neuroprotection. Hormones and Behavior, 63, 284–290. 22732134 10.1016/j.yhbeh.2012.06.003 PMC3467329

[hbm26184-bib-0059] Small, D. M. , & Green, B. G. (2012). A proposed model of a flavor modality. In M. M. Murray & M. T. Wallace (Eds.), The neural bases of multisensory processes. CRC Press/Taylor & Francis. Frontiers in Neuroscience.

[hbm26184-bib-0060] Spreng, R. N. , Stevens, W. D. , Viviano, J. D. , & Schacter, D. L. (2016). Attenuated anticorrelation between the default and dorsal attention networks with aging: Evidence from task and rest. Neurobiology of Aging, 45, 149–160. 27459935 10.1016/j.neurobiolaging.2016.05.020 PMC5003045

[hbm26184-bib-0061] Stöckel, T. , Wunsch, K. , & Hughes, C. M. L. (2017). Age‐related decline in anticipatory motor planning and its relation to cognitive and motor skill proficiency. Frontiers in Aging Neuroscience, 9, 283. 28928653 10.3389/fnagi.2017.00283 PMC5591340

[hbm26184-bib-0062] Stoodley, C. J. , & Schmahmann, J. D. (2009). Functional topography in the human cerebellum: A meta‐analysis of neuroimaging studies. NeuroImage, 44, 489–501. 18835452 10.1016/j.neuroimage.2008.08.039

[hbm26184-bib-0063] Stoodley, C. J. , Valera, E. M. , & Schmahmann, J. D. (2012). Functional topography of the cerebellum for motor and cognitive tasks: An fMRI study. NeuroImage, 59, 1560–1570. 21907811 10.1016/j.neuroimage.2011.08.065 PMC3230671

[hbm26184-bib-0064] Taylor, J. R. , Williams, N. , Cusack, R. , Auer, T. , Shafto, M. A. , Dixon, M. , Tyler, L. K. , & Cam‐Can, H. R. N. (2017). The Cambridge Centre for Ageing and Neuroscience (Cam‐CAN) data repository: Structural and functional MRI, MEG, and cognitive data from a cross‐sectional adult lifespan sample. NeuroImage, 144, 262–269. 26375206 10.1016/j.neuroimage.2015.09.018 PMC5182075

[hbm26184-bib-0065] Van Dijk, K. R. A. , Sabuncu, M. R. , & Buckner, R. L. (2012). The influence of head motion on intrinsic functional connectivity MRI. NeuroImage, 59, 431–438. 21810475 10.1016/j.neuroimage.2011.07.044 PMC3683830

[hbm26184-bib-0066] Vossel, S. , Geng, J. J. , & Fink, G. R. (2014). Dorsal and ventral attention systems: Distinct neural circuits but collaborative roles. The Neuroscientist, 20, 150–159. 23835449 10.1177/1073858413494269 PMC4107817

[hbm26184-bib-0067] Wallis, G. , Stokes, M. , Cousijn, H. , Woolrich, M. , & Nobre, A. C. (2015). Frontoparietal and cingulo‐opercular networks play dissociable roles in control of working memory. Journal of Cognitive Neuroscience, 27, 2019–2034. 26042457 10.1162/jocn_a_00838

[hbm26184-bib-0068] Whitfield‐Gabrieli, S. , & Nieto‐Castanon, A. (2012). Conn: A functional connectivity toolbox for correlated and anticorrelated brain networks. Brain Connectivity, 2, 125–141. 22642651 10.1089/brain.2012.0073

[hbm26184-bib-0069] Wig, G. S. (2017). Segregated systems of human brain networks. Trends in Cognitive Sciences, 21, 981–996. 29100737 10.1016/j.tics.2017.09.006

[hbm26184-bib-0070] Wolfson, L. , Whipple, R. , Derby, C. A. , Amerman, P. , & Nashner, L. (1994). Gender differences in the balance of healthy elderly as demonstrated by dynamic posturography. Journal of Gerontology, 49, M160–M167. 8014390 10.1093/geronj/49.4.m160

[hbm26184-bib-0071] Zar, J. H. (1996). Biostatistical analysis. Prentice Hall.

