# Supplementary material for: Age‐related differences in functional network segregation in the context of sex and reproductive stage
Source: Hum Brain Mapp. 2022 Dec 21;44(5):1949–63. doi: 10.1002/hbm.26184 (PMC9980887; doi:10.1002/hbm.26184)
Supplement: Supplementary file 1 — Appendix S1: Supporting information. [file HBM-44-1949-s002.docx]

**Supplementary Results**

The following text, and associated tables and figures, depict results from an exploratory analysis where a more stringent motion correction threshold of 0.2 mm was applied in place of the conservative 0.5 mm threshold used in the main text. Otherwise, our approach from the main text was replicated here using this modified threshold.

For context, we present two figures below illustrating relationships between age and average mean motion observed (**Figure S1**) as well as between age and average maximum motion observed (**Figure S2**) across our final sample of 414 subjects. Notably, age was negatively correlated with mean motion (*p* < 0.001; *r* = -0.44); thus, young adults moved more than the older adults within our sample. In addition, age was negatively correlated with maximum motion (*p* < 0.001; *r* = -0.25), corroborating that our younger subjects had greater motion in the scanner, relative to older adults. For mean motion, the average across our sample was 0.095 mm, and the average maximum motion across subjects was 0.489 mm. Maximum motion points that were above the modified 0.2 mm threshold for motion correction were subsequently covaried-out during preprocessing for the analysis presented below.

**Figure S1**. Mean motion by age for the final cohort of 414 subjects, after applying a stringent 0.2 mm motion correction threshold (*p* < 0.001; *r* = -0.44).

**Figure S2.** Maximum motion by age for the final cohort of 414 subjects, using the 0.2 mm threshold for motion correction (*p* < 0.001; *r* = -0.25).

*Outlier Exclusions*

For the ventral attention network, one extreme outlier with a segregation value of -540.94, corresponding to 326 standard deviations (SD) below the original network mean, was removed. To fairly screen for true outliers in the ventral attention network, this extreme outlier was removed before performing subsequent exclusions. Values above or below three SDs from the adjusted network mean, after removing the extreme outlier, were also excluded as outliers for the ventral attention network. In addition, two extreme outliers were detected and preemptively removed from the dorsal attention network prior to subsequent outlier exclusions: one value of 630.06 (1,491 SDs above the network mean) and a value of -87.34 (209 SDs below the mean).

*Age Correlations*

Detailed results for network segregation and age correlations are reported in **Table S1**. When considering age and mean network segregation (computed across all networks), there is a significant correlation (*p* = 0.038) such that increased age is associated with lower overall network segregation across participants; however, this correlation did not survive multiple comparisons correction with a False Discovery Rate (FDR) approach (**Figure S3; Table S1**). Considering each network individually, a significant correlation between age and network segregation emerges only for the fronto-parietal task control network after applying FDR corrections (*p* = 0.024). A significant correlation between age and segregation was also present for the salience network but did not remain significant after FDR correction. Each of these individual network correlations indicate lower network segregation with increased age (**Figure S3; Table S1**).

| Network | T | Df | P_raw_ | P_fdr_ | R |
| --- | --- | --- | --- | --- | --- |
| Whole Sample | | | | | |
| All Networks | -2.08 | 401 | *0.038 | 0.152 | -0.10 |
| Auditory | 0.83 | 404 | 0.409 | - | 0.04 |
| Cerebellar-Basal Ganglia | 1.05 | 410 | 0.296 | - | 0.05 |
| Cingulo-Opercular Task Control | -1.10 | 409 | 0.271 | - | -0.05 |
| Default Mode | -0.92 | 401 | 0.357 | - | -0.05 |
| Dorsal Attention | -0.58 | 404 | 0.560 | - | -0.03 |
| Fronto-Parietal Task Control | -3.16 | 408 | **0.002 | *0.024 | -0.15 |
| Salience | -2.32 | 406 | *0.021 | 0.126 | -0.11 |
| Sensory Somatomotor Hand | 0.59 | 404 | 0.558 | - | 0.03 |
| Sensory Somatomotor Mouth | 0.11 | 406 | 0.912 | - | 0.01 |
| Ventral Attention | 0.81 | 407 | 0.416 | - | 0.04 |
| Visual | -1.42 | 405 | 0.155 | - | -0.07 |

**Table S1.** *Pearson’s correlations between age and network segregation across the whole sample*. P_raw_: un-corrected p values; P_fdr_: adjusted p values after FDR multiple comparisons corrections. *: *p* < .05; **: *p* < .01; ***: *p* < .001.

**Figure S3.** *Relationships between age and network segregation across all participants*. Distributions for each individual network and the mean across all networks. Each plot includes a linear regression line and the associated correlation value. *****: *p* < .05). The ventral attention network was plotted on a separate scale from all remaining networks for interpretability.

Correlations in females only do not reveal any significant age-segregation associations after multiple comparisons corrections (**Figure S4; Table S2**). However, prior to FDR corrections, and similar to the whole-sample results, females show a negative correlation between age and fronto-parietal task control segregation, though significance is reduced to trending (*p* = 0.072) after correcting for multiple comparisons.

| Network | T | Df | P_raw_ | P_fdr_ | R |
| --- | --- | --- | --- | --- | --- |
| Females Only | | | | | |
| All Networks | -0.90 | 199 | 0.367 | - | -0.06 |
| Auditory | -0.10 | 199 | 0.920 | - | -0.01 |
| Cerebellar-Basal Ganglia | 0.85 | 205 | 0.399 | - | 0.06 |
| Cingulo-Opercular Task Control | -1.28 | 203 | 0.202 | - | -0.09 |
| Default Mode | -0.42 | 199 | 0.675 | - | -0.03 |
| Dorsal Attention | 1.36 | 200 | 0.175 | - | 0.10 |
| Fronto-Parietal Task Control | -2.78 | 202 | **0.006 | 0.072 | -0.19 |
| Salience | -1.17 | 202 | 0.242 | - | -0.08 |
| Sensory Somatomotor Hand | 1.07 | 202 | 0.286 | - | 0.08 |
| Sensory Somatomotor Mouth | 0.48 | 198 | 0.634 | - | 0.03 |
| Ventral Attention | 0.77 | 202 | 0.441 | - | 0.05 |
| Visual | -1.13 | 201 | 0.260 | - | -0.08 |
| Males Only | | | | | |
| All Networks | -2.10 | 200 | *0.037 | 0.148 | -0.15 |
| Auditory | 1.34 | 203 | 0.182 | - | 0.09 |
| Cerebellar-Basal Ganglia | 0.62 | 203 | 0.536 | - | 0.04 |
| Cingulo-Opercular Task Control | -0.15 | 204 | 0.878 | - | -0.01 |
| Default Mode | -0.90 | 200 | 0.368 | - | -0.06 |
| Dorsal Attention | -2.18 | 202 | *0.030 | 0.148 | -0.15 |
| Fronto-Parietal Task Control | -1.64 | 204 | 0.103 | - | -0.11 |
| Salience | -2.14 | 202 | *0.033 | 0.148 | -0.15 |
| Sensory Somatomotor Hand | -0.23 | 200 | 0.821 | - | -0.02 |
| Sensory Somatomotor Mouth | -0.41 | 203 | 0.679 | - | -0.03 |
| Ventral Attention | 0.36 | 203 | 0.718 | - | 0.03 |
| Visual | -0.86 | 202 | 0.389 | - | -0.06 |

**Table S2.** *Pearson’s correlations between age and network segregation within each sex separately*. *: *p* < .05; **: *p* < .01; ***: *p* < .001.

**Figure S4.** *Relationships between age and network segregation, separated by sex*. Distributions for each individual network and the mean across all networks. Females are plotted in purple and males in blue. Each plot includes linear regression lines and associated correlation values. F: females; M: males; *: *p* < .05. The ventral attention network was plotted on a separate scale from all remaining networks for interpretability.

In age-matched male controls, we also do not observe any significant age-segregation associations after correcting for multiple comparisons (**Figure S4; Table S2**). Prior to applying FDR corrections, male controls demonstrate significant associations between age and network segregation across all networks and within two individual networks: dorsal attention and salience, though these correlations do not survive multiple comparisons corrections. Overall, it seems that a few sex-specific differences in functional network segregation declines with age are present before FDR corrections, but none of these differences subsequently remain significant.

To expand upon the correlational results and test potential interactions between sex and age, we then performed a series of ANCOVAs (**Table S3**). Each ANCOVA included sex and age, as well as the interaction between these variables, as factors with respect to network segregation. In brief, we found a main effect of age for the fronto-parietal task control network after applying FDR correction (*p* = 0.048), which parallels the whole-sample results. A significant effect of sex and an interaction between sex and age was also observed for the dorsal attention network, though neither effect survived multiple comparisons corrections.

| Effect | Dfn | Dfd | F | P_raw_ | P_fdr_ | Effect Size |
| --- | --- | --- | --- | --- | --- | --- |
| Sex (Female vs Male) X Age ANCOVAs | | | | | | |
| *All Networks* | | | | | | |
| Age | 1 | 399 | 0.88 | 0.349 | - | 0.01 |
| Sex | 1 | 399 | 1.51 | 0.219 | - | 0.02 |
| Age:Sex | 1 | 399 | 0.59 | 0.445 | - | 0.01 |
| *Auditory* | | | | | | |
| Age | 1 | 402 | 0.01 | 0.917 | - | 0.00 |
| Sex | 1 | 402 | 0.33 | 0.567 | - | 0.01 |
| Age:Sex | 1 | 402 | 0.95 | 0.331 | - | 0.03 |
| *Cerebellar-Basal Ganglia* | | | | | | |
| Age | 1 | 408 | 0.85 | 0.356 | - | 0.06 |
| Sex | 1 | 408 | 0.24 | 0.626 | - | 0.02 |
| Age:Sex | 1 | 408 | 0.07 | 0.795 | - | 0.00 |
| *Cingulo-Opercular Task Control* | | | | | | |
| Age | 1 | 407 | 2.03 | 0.155 | - | 0.07 |
| Sex | 1 | 407 | 0.35 | 0.555 | - | 0.01 |
| Age:Sex | 1 | 407 | 0.84 | 0.361 | - | 0.03 |
| *Default Mode* | | | | | | |
| Age | 1 | 399 | 0.19 | 0.663 | - | 0.01 |
| Sex | 1 | 399 | 0.13 | 0.718 | - | 0.01 |
| Age:Sex | 1 | 399 | 0.09 | 0.760 | - | 0.00 |
| *Dorsal Attention* | | | | | | |
| Age | 1 | 402 | 1.83 | 0.177 | - | 0.08 |
| Sex | 1 | 402 | 6.95 | **0.009 | 0.108 | 0.32 |
| Age:Sex | 1 | 402 | 6.30 | *0.012 | 0.144 | 0.29 |
| *Fronto-Parietal Task Control* | | | | | | |
| Age | 1 | 406 | 8.39 | **0.004 | *0.048 | 0.43 |
| Sex | 1 | 406 | 0.64 | 0.425 | - | 0.03 |
| Age:Sex | 1 | 406 | 0.89 | 0.345 | - | 0.05 |
| *Salience* | | | | | | |
| Age | 1 | 404 | 1.46 | 0.227 | - | 0.06 |
| Sex | 1 | 404 | 0.47 | 0.491 | - | 0.02 |
| Age:Sex | 1 | 404 | 0.37 | 0.542 | - | 0.02 |
| *Sensory Somatomotor Hand* | | | | | | |
| Age | 1 | 402 | 1.11 | 0.293 | - | 0.02 |
| Sex | 1 | 402 | 2.02 | 0.156 | - | 0.03 |
| Age:Sex | 1 | 402 | 0.82 | 0.366 | - | 0.01 |
| *Sensory Somatomotor Mouth* | | | | | | |
| Age | 1 | 401 | 0.27 | 0.606 | - | 0.00 |
| Sex | 1 | 401 | 1.29 | 0.256 | - | 0.01 |
| Age:Sex | 1 | 401 | 0.40 | 0.527 | - | 0.00 |
| *Ventral Attention* | | | | | | |
| Age | 1 | 405 | 0.65 | 0.421 | - | 0.14 |
| Sex | 1 | 405 | 0.29 | 0.589 | - | 0.06 |
| Age:Sex | 1 | 405 | 0.11 | 0.742 | - | 0.02 |
| *Visual* | | | | | | |
| Age | 1 | 403 | 1.44 | 0.232 | - | 0.03 |
| Sex | 1 | 403 | 0.46 | 0.499 | - | 0.01 |
| Age:Sex | 1 | 403 | 0.08 | 0.781 | - | 0.00 |

**Table S3.** *ANCOVA results for sex-age interactions*. *: *p* < .05; **: *p* < .01; ***: *p* < .001.

*Reproductive Stage Comparisons*

An overview of reproductive stage and sex effects on network segregation is provided in **Table S4** and depicted in **Figure S5**. An effect of stage (reproductive vs. late postmenopausal/relative controls) was significant for only the fronto-parietal task control network after applying multiple comparisons corrections (*p* = 0.002); however, an effect of stage was also present for the mean across all networks prior to FDR corrections. Notably, a significant effect of sex (females vs. males) was observed with the visual network but was reduced to a trending significance level (*p* = 0.084) after multiple comparisons correction. In addition, an interaction between reproductive stage and sex was significant for the dorsal attention network and survived FDR corrections (*p* = 0.048). Given the general lack of significant effects, this series of analyses suggests that reproductive stage within females does not differentially impact functional network segregation beyond the broader impacts of age for most of the networks considered here.

| Effect | Dfn | Dfd | F | P_raw_ | P_fdr_ | Effect Size |
| --- | --- | --- | --- | --- | --- | --- |
| 2 (Reproductive vs Late Postmenopausal) X 2 (Female vs Male) ANOVAs | | | | | | |
| *All Networks* | | | | | | |
| Stage | 1 | 327 | 4.57 | *0.033 | 0.198 | 0.01 |
| Sex | 1 | 327 | 2.16 | 0.142 | - | 0.01 |
| Stage:Sex | 1 | 327 | 0.40 | 0.527 | - | 0.00 |
| *Auditory* | | | | | | |
| Stage | 1 | 327 | 0.40 | 0.530 | - | 0.00 |
| Sex | 1 | 327 | 0.39 | 0.533 | - | 0.00 |
| Stage:Sex | 1 | 327 | 2.05 | 0.153 | - | 0.01 |
| *Cerebellar-Basal Ganglia* | | | | | | |
| Stage | 1 | 332 | 1.24 | 0.265 | - | 0.00 |
| Sex | 1 | 332 | 0.00 | 0.949 | - | 0.00 |
| Stage:Sex | 1 | 332 | 0.00 | 0.960 | - | 0.00 |
| *Cingulo-Opercular Task Control* | | | | | | |
| Stage | 1 | 332 | 1.33 | 0.250 | - | 0.00 |
| Sex | 1 | 332 | 0.23 | 0.629 | - | 0.00 |
| Stage:Sex | 1 | 332 | 1.13 | 0.289 | - | 0.00 |
| *Default Mode* | | | | | | |
| Stage | 1 | 327 | 2.05 | 0.154 | - | 0.01 |
| Sex | 1 | 327 | 0.54 | 0.464 | - | 0.00 |
| Stage:Sex | 1 | 327 | 0.29 | 0.587 | - | 0.00 |
| *Dorsal Attention* | | | | | | |
| Stage | 1 | 327 | 0.85 | 0.359 | - | 0.00 |
| Sex | 1 | 327 | 1.35 | 0.246 | - | 0.00 |
| Stage:Sex | 1 | 327 | 8.39 | **0.004 | *0.048 | 0.03 |
| *Fronto-Parietal Task Control* | | | | | | |
| Stage | 1 | 331 | 13.98 | ***< 0.001 | **0.002 | 0.04 |
| Sex | 1 | 331 | 0.08 | 0.782 | - | 0.00 |
| Stage:Sex | 1 | 331 | 0.50 | 0.478 | - | 0.00 |
| *Salience* | | | | | | |
| Stage | 1 | 330 | 3.76 | 0.053 | - | 0.01 |
| Sex | 1 | 330 | 0.08 | 0.782 | - | 0.00 |
| Stage:Sex | 1 | 330 | 0.03 | 0.871 | - | 0.00 |
| *Sensory Somatomotor Hand* | | | | | | |
| Stage | 1 | 327 | 0.03 | 0.858 | - | 0.00 |
| Sex | 1 | 327 | 1.12 | 0.291 | - | 0.00 |
| Stage:Sex | 1 | 327 | 0.18 | 0.671 | - | 0.00 |
| *Sensory Somatomotor Mouth* | | | | | | |
| Stage | 1 | 328 | 0.00 | 0.993 | - | 0.00 |
| Sex | 1 | 328 | 1.04 | 0.309 | - | 0.00 |
| Stage:Sex | 1 | 328 | 0.31 | 0.578 | - | 0.00 |
| *Ventral Attention* | | | | | | |
| Stage | 1 | 329 | 0.37 | 0.542 | - | 0.00 |
| Sex | 1 | 329 | 0.46 | 0.499 | - | 0.00 |
| Stage:Sex | 1 | 329 | 0.00 | 0.961 | - | 0.00 |
| *Visual* | | | | | | |
| Stage | 1 | 329 | 3.10 | 0.079 | - | 0.01 |
| Sex | 1 | 329 | 7.44 | **0.007 | 0.084 | 0.02 |
| Stage:Sex | 1 | 329 | 0.08 | 0.781 | - | 0.00 |

**Table S4**. *Between-subjects ANOVA results*. *: *p* < .05; **: *p* < .01; ***: *p* < .001.

**Figure S5.** *Network segregation by stage (reproductive vs. late postmenopausal/relative controls) and sex (female vs. male).* Average segregation value per group for individual networks and the mean across all networks. Error bars depict standard error. ALL: Mean across all networks; AU: Auditory; CBBG: Cerebellar-basal ganglia; COTC: Cingulo-opercular task control; DM: Default mode; DA: Dorsal attention; FPTC: Fronto-parietal task control; SA: Salience; SSH: Sensory somatomotor hand; SSM: Sensory somatomotor mouth; VA: Ventral attention; VI: Visual.
